# Supplementary material for: Resolving the full spectrum of human genome variation using Linked-Reads
Source: Genome Res. 2019 Apr;29(4):635–45. doi: 10.1101/gr.234443.118 (PMC6442396; doi:10.1101/gr.234443.118)
Supplement: Supplemental Material [file supp_gr.234443.118_Supplemental_longranger-2.2.zip › longranger-2.2.tar.bz2!/lr2.2-public/lib/stan/cmdstan/stan/doc/api/html/structstan_1_1math_1_1promote__scalar__struct_3_01_t_00_01std_1_1vector_3_01_s_01_4_01_4.html]

Stan: stan::math::promote\_scalar\_struct< T, std::vector< S > > Struct Template Reference


|  |  |
| --- | --- |
|  | Stan  2.5.0  probability, sampling & optimization |


- Main Page
- Related Pages
- Namespaces
- Classes
- Files

- Class List
- Class Index
- Class Hierarchy
- Class Members

All Classes Namespaces Files Functions Variables Typedefs Enumerations Enumerator Friends Macros Pages

- stan
- math
- promote\_scalar\_struct< T, std::vector< S > >

Static Public Member Functions |
List of all members

stan::math::promote\_scalar\_struct< T, std::vector< S > > Struct Template Reference

Struct to hold static function for promoting underlying scalar types.
More...

`#include <promote_scalar.hpp>`

|  |  |
| --- | --- |
| Static Public Member Functions | |
| static std::vector< typename   promote\_scalar\_type< T, S >  ::type > | apply (const std::vector< S > &x) |
|  | Return the standard vector consisting of the recursive promotion of the elements of the input standard vector to the scalar type specified by the return template parameter. More... |
|  | |

## Detailed Description

### template<typename T, typename S> struct stan::math::promote\_scalar\_struct< T, std::vector< S > >

Struct to hold static function for promoting underlying scalar types.

This specialization is for standard vector inputs.

Template Parameters
:   |  |  |
    | --- | --- |
    | T | return scalar type |
    | S | input type for standard vector elements in static nested function, which must have an underlying scalar type assignable to T. |

Definition at line 70 of file promote\_scalar.hpp.

## Member Function Documentation

template<typename T , typename S >

|  |  |  |  |  |  |
| --- | --- | --- | --- | --- | --- |
| static std::vector<typename promote\_scalar\_type<T,S>::type> stan::math::promote\_scalar\_struct< T, std::vector< S > >::apply | ( | const std::vector< S > & | *x* | ) |  |

inlinestatic

Return the standard vector consisting of the recursive promotion of the elements of the input standard vector to the scalar type specified by the return template parameter.

Parameters
:   |  |  |
    | --- | --- |
    | x | input standard vector. |

Returns
:   standard vector with values promoted from input vector.

Definition at line 81 of file promote\_scalar.hpp.

---

The documentation for this struct was generated from the following file:

- src/stan/math/functions/promote\_scalar.hpp


---

[ Stan Home Page ]

*© 2011–2014,
Stan Development Team.*
